# An ensemble deep learning diagnostic system for determining Clinical Activity Scores in thyroid-associated ophthalmopathy: integrating multi-view multimodal images from anterior segment slit-lamp photographs and facial images

**DOI:** 10.3389/fendo.2024.1365350

**Published:** 2024-04-02

**Authors:** Chunfang Yan, Zhaoxia Zhang, Guanghua Zhang, Han Liu, Ruiqi Zhang, Guiqin Liu, Jing Rao, Weihua Yang, Bin Sun

**Affiliations:** ^1^ Shanxi Eye Hospital Affiliated to Shanxi Medical University, Taiyuan, Shanxi, China; ^2^ School of Big Data Intelligent Diagnosis and Treatment Industry, Taiyuan University, Taiyuan, Shanxi, China; ^3^ College of Computer Science and Technology, Taiyuan Normal University, Taiyuan, Shanxi, China; ^4^ Shenzhen Eye Institute, Shenzhen Eye Hospital, Jinan University, Shenzhen, Guangdong, China

**Keywords:** thyroid-associated ophthalmopathy, ensemble deep learning, multi-view multimodal, clinical activity score, active TAO diagnosis

## Abstract

**Background:**

Thyroid-associated ophthalmopathy (TAO) is the most prevalent autoimmune orbital condition, significantly impacting patients’ appearance and quality of life. Early and accurate identification of active TAO along with timely treatment can enhance prognosis and reduce the occurrence of severe cases. Although the Clinical Activity Score (CAS) serves as an effective assessment system for TAO, it is susceptible to assessor experience bias. This study aimed to develop an ensemble deep learning system that combines anterior segment slit-lamp photographs of patients with facial images to simulate expert assessment of TAO.

**Method:**

The study included 156 patients with TAO who underwent detailed diagnosis and treatment at Shanxi Eye Hospital Affiliated to Shanxi Medical University from May 2020 to September 2023. Anterior segment slit-lamp photographs and facial images were used as different modalities and analyzed from multiple perspectives. Two ophthalmologists with more than 10 years of clinical experience independently determined the reference CAS for each image. An ensemble deep learning model based on the residual network was constructed under supervised learning to predict five key inflammatory signs (redness of the eyelids and conjunctiva, and swelling of the eyelids, conjunctiva, and caruncle or plica) associated with TAO, and to integrate these objective signs with two subjective symptoms (spontaneous retrobulbar pain and pain on attempted upward or downward gaze) in order to assess TAO activity.

**Results:**

The proposed model achieved 0.906 accuracy, 0.833 specificity, 0.906 precision, 0.906 recall, and 0.906 F1-score in active TAO diagnosis, demonstrating advanced performance in predicting CAS and TAO activity signs compared to conventional single-view unimodal approaches. The integration of multiple views and modalities, encompassing both anterior segment slit-lamp photographs and facial images, significantly improved the prediction accuracy of the model for TAO activity and CAS.

**Conclusion:**

The ensemble multi-view multimodal deep learning system developed in this study can more accurately assess the clinical activity of TAO than traditional methods that solely rely on facial images. This innovative approach is intended to enhance the efficiency of TAO activity assessment, providing a novel means for its comprehensive, early, and precise evaluation.

## 1 Introduction

Thyroid-associated ophthalmopathy (TAO), also known as thyroid eye disease, is an organ-specific autoimmune disease that is closely related to thyroid diseases. It is the most common cause of adult orbital disease and also the most common extrathyroidal manifestation of diffuse toxic goiter (Graves’ disease, GD) Bartalena et al. (1). The clinical manifestations of TAO are diverse and complex, including unilateral or bilateral eyelid retraction, exophthalmos, diplopia, restrictive strabismus, exposure keratopathy, and dysthyroid optic neuropathy, all of which significantly affect a patient’s quality of life Sun et al. (2).

Accurate assessment of TAO’s activity is indispensable for effective management, with active disease responsive to immunotherapy and inactive disease often marked by persistent fibrotic changes Bartalena et al. (3), Dong et al. (4). The Clinical Activity Score (CAS), a seven-item scale reflecting disease manifestations like redness, swelling, and retrobulbar pain, is pivotal for gauging disease activity Mourits et al. (5). However, consistency in CAS application depends on the clinician’s expertise, highlighting the necessity for a more objective assessment method Bartalena et al. (6), Wang et al. (7).

The advent of artificial intelligence (AI) and deep learning has provided new prospects for medical diagnostics, including ophthalmology Yoo and Choi (8), Benet and Pellicer-Valero (9), Chen et al. (10), Gong et al. (11). Despite this progress, the current AI models for TAO activity assessment have notable limitations. These models typically rely on unimodal single-view imaging and primarily use facial images Huang et al. (12), Moon et al. (13), Karlin et al. (14). Studies Abboud et al. (15), Vokurka Topljak et al. (16) have indicated that a unimodal approach can lead to significant oversight of crucial disease markers due to its inability to capture the heterogeneity and three-dimensional aspects of various pathologies. Multi-view imaging enables the capture of a disease manifestation from various angles, thus providing a richer and more complete representation. It is crucial especially in the case of TAO, where the orbital inflammation might not be uniformly distributed or may present subtle variations that are perceptible only from specific angles or through particular imaging techniques.

**Figure 1:**
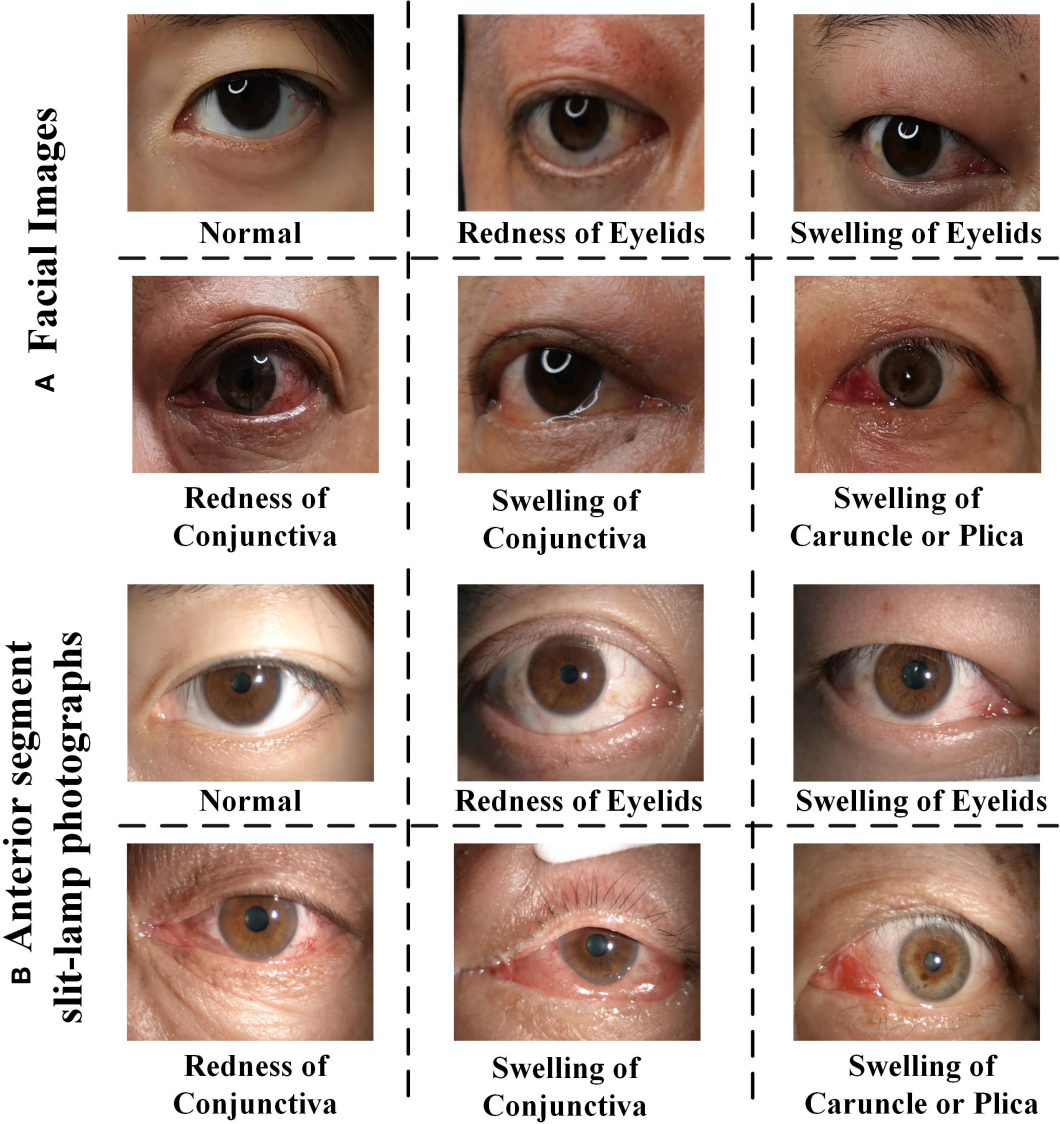
The representative samples of RE (Redness of Eyelid), SE (Swelling of Eyelids), RC (Redness of Conjunctiva), SC (Swelling of Conjunctiva), SCP (Swelling of Caruncle or Plica), and normal eyes. **(A)** Face images, **(B)** anterior segment slit-lamp photographs. Note that each positive image may contains multiple TAO signs.

Furthermore, the integration of different modalities such as anterior segment slit-lamp photography, often used to closely examine the eyes, with traditional facial photographs, allows for a more robust and holistic assessment. The representative samples in multi-view multimodal forms are illustrated in **Figure 1**. The drawbacks of relying on single-view facial photos—such as the potential to miss oblique or lateral pathological signs, the challenges posed by shadowing effects, and the difficulty in assessing three-dimensional structures—are significant. Therefore, it is essential to employ facial photographs from multiple angles, ensuring a more homogeneous and precise evaluation that is not overly dependent on the expertise or subjective interpretation of the clinician.

Thus, previous AI machine learning-assisted systems that utilized single-modality digital facial images to determine the CAS of TAO often failed to consider swelling of the conjunctiva and of the caruncle or plica Lu et al. (17), Gu et al. (18), Li et al. (19). The failure of existing AI models to include these perspectives has resulted in partial understanding of the disease, missing the intricate interplay of symptoms and signs that is crucial for accurate diagnosis. Moreover, these models often lack integration of clinical context, which is a key aspect in the accurate diagnosis and management of TAO. The absence of anterior segment slit-lamp photographs in these models underscores a significant gap in the diagnostic process, highlighting the urgent need for a more inclusive and comprehensive AI system that integrates multimodal and multi-view data to assess CAS in patients with TAO.

To address these limitations, we developed an ensemble deep learning (EnsembleDL) system in this study. This innovative system integrates multiple perspectives and modalities, including anterior segment slit-lamp photographs and facial images taken from various angles (such as the upper, lower, left, right, and frontal views of facial images, combined with frontal and lateral views of the anterior segments of both eyes). The inclusion of slit-lamp images is particularly crucial as they enable clear observation of swellings of the conjunctiva and caruncle or plica region for accurate scoring. We also evaluated the accuracy of the EnsembleDL system in predicting CAS and diagnosing active TAO, while exploring its potential as a screening tool for assessing TAO activity.

## 2 Material and methods

**Figure 2:**
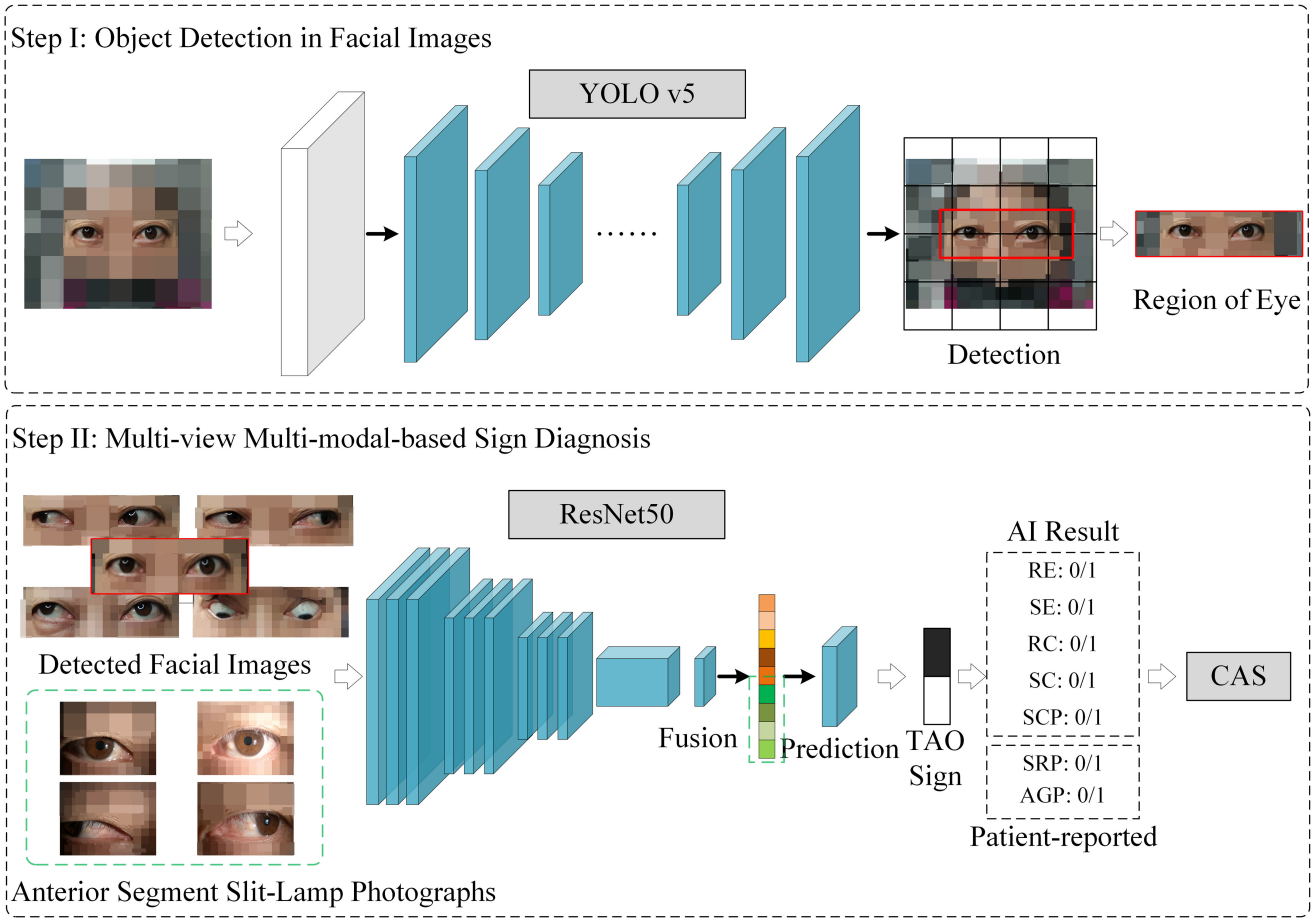
The multi-view multimodal deep learning system for thyroid-associated ophthalmopathy sign prediction and clinical activity score calculation. RE (Redness of Eyelids), SE (Swelling of Eyelids), RC (Redness of Conjunctiva), SC (Swelling of Conjunctiva), SCP (Swelling of Caruncle or Plica), SRP (Spontaneous Retrobulbar Pain), AGP (Attempted Upward or Downward Gaze Pain).

This study integrated multiple views and modalities to predict CASMourits et al. (20) in TAO, using data collected from 156 patients at Shanxi Eye Hospital Affiliated to Shanxi Medical University, from May 2020 to September 2023. The dataset, featuring both anterior-segment slit-lamp and facial images, lays the groundwork for our novel AI diagnostic approach. Using an ensemble ResNet50-based model for intricate feature extraction and subsequent feature fusion processes, we enhanced the multidimensional analysis of TAO activity, surpassing traditional single-view unimodal diagnostic methods. The overall method is illustrated in **Figure 2**.

The study was approved by the Ethics Review Committee of the Shanxi Eye Hospital Affiliated to Shanxi Medical University (SXYYLL-20230202KS). Owing to the retrospective design of the study and the use of past medical records and facial images, the ethics review committee waived the requirement for informed consent. All experiments were conducted in accordance with the relevant guidelines and regulations. All images and clinical data were anonymized before external processing, and the data transfer process was approved by the ethics review committee.

### 2.1 Data acquisition

#### 2.1.1 Imaging and diagnosis

All facial images from the study subjects were captured at a distance of 25 cm, using a smartphone (model number of Honor 20 Pro) under 2340x1080 pixels. The utilized mobile phone is equipped with a 48-megapixel rear main camera (aperture f/1.4), allowing for high-quality image acquisition with support for various photography modes. The phone was held steady by a specialized phone stand, to ensure consistent and stable positioning for photography. Patients’ diagnoses were extracted from the outpatient and inpatient medical records. None of the patients had ocular comorbidities that influenced their facial expressions or images. Five gaze positions were photographed from the front, namely primary, upper, lower, left, and right. The patients were instructed to keep their heads steady with their eyes primarily positioned, and their head positions were checked to confirm the absence of apparent tilting or upward or downward chin movements. Anterior segment slit-lamp photographs of all the patients were obtained using a slit lamp (CSO SL990N, 2448x2048 pixels; Florence, Italy) at a magnification of 10 ×. Both eyes of each patient were photographed in primary and lateral positions, fully exposing the caruncle and plica.

Besides, regarding the variability of illumination conditions during the capture of slit-lamp photographs, which may potentially affect the consistency of Clinical Activity Score (CAS) evaluations, we took several steps to standardize the lighting conditions during image acquisition. Our slit-lamp imaging protocol adhered strictly to clinical standards, where the intensity and angle of the illuminator were maintained consistently across all examinations. This protocol was followed meticulously to minimize variations in lighting conditions and ensure uniform image quality for CAS evaluations. Moreover, recognizing that slight variations in lighting are inevitable, our AI model was specifically designed to be robust against such fluctuations. Advanced image processing techniques were implemented to normalize the images before they were presented to the deep learning model. Furthermore, the deep learning model was trained on a diverse dataset that included images with a range of lighting conditions to improve its generalizability and robustness. This ensures that the model can recognize and assess the key inflammatory signs of TAO in a consistent manner, independent of minor variations in illumination.

#### 2.1.2 Determination of the reference CAS

In establishing the reference CAS, our process adhered to a standardized assessment protocol. Two experienced ophthalmologists, with more than 10 years of clinical experience, utilized both the image data and clinical examination findings of patients to simulate a realistic clinical environment and ensure an accurate evaluation. To guarantee consistency in scoring, both ophthalmologists underwent joint training and calibration sessions for the CAS assessment prior to scoring.

In instances where the two ophthalmologists’ CAS scores differed, we employed the following strategy to determine the final reference CAS: An adjudication meeting was convened where both ophthalmologists re-evaluated the images and clinical data, discussing the scores until consensus was reached. If discordance persisted post-adjudication meeting, a third, senior ophthalmologist’s expertise was sought, and their scoring was adopted as the final reference CAS.

#### 2.1.3 Inclusion and exclusion criteria

The criteria for selecting patients for this study were carefully designed to ensure the reliability of the data and the accuracy of the CAS assessment in TAO. The inclusion criteria were as follows:

1) Definitive diagnosis of TAO with stable, high-quality images and complete case data: This ensures the accuracy and consistency of the data set. High-quality images are crucial for the precise identification of key inflammatory signs in TAO.
2) Disease duration not exceeding 18 months: The focus of this study is on the active phase of TAO, which is typically observed within the first 18 months of disease onset. During this stage, pharmacotherapy usually shows suboptimal effects, and patients with severe conditions require surgical interventions. This study aims to identify patients with early active TAO to guide them toward proactive pharmacological interventions, with the objective of preventing the exacerbation of the condition.
3) Controlled thyroid function: Patients with uncontrolled thyroid function can exhibit fluctuating symptoms, which can confound the assessment of TAO activity. Controlled thyroid function provides a more stable context for evaluating TAO independent of thyroid hormone fluctuations.
4) No history of radiation therapy, surgical decompression, or other immunosuppressive treatments: These treatments can significantly alter the clinical presentation and progression of TAO, thereby affecting the assessment of disease activity and the CAS.
5) Absence of complications posing a threat to vision and no other orbital diseases: For patients with severe TAO posing a threat to vision, immediate and timely treatment is crucial to maximize the preservation of the patient’s eyesight. This criterion ensures that any changes in the CAS are directly related to TAO and not influenced by other ocular or orbital pathologies.

Patients with incomplete medical records or unclear diagnoses were excluded, as these factors could lead to unreliable CAS assessments. Additionally, patients with unstable thyroid function were excluded, as their fluctuating symptoms could confound the objective assessment of TAO activity. Besides, we also introduce several normal samples in the model training.

### 2.2 Eye region detection in facial photographs

To effectively process the multi-view facial images of patients with TAO, we first developed a specialized YOLO V5 model Redmon et al. (21) with the backbone net of CSP-Darknet Bochkovskiy et al. (22) to detect eye regions in facial images. Each facial image was initially annotated according to the eye region. Raw facial images along with their annotations were then fed into the YOLO V5 model. To ensure uniformity, all images were resized to 640×640 pixels. This process involved 5×156 = 780 facial images. For model training and evaluation, we divided the dataset into training and testing sets in an 8:2 ratio and set the learning rate to 0.01 along with the SGD optimizer Kiefer and Wolfowitz (23). The YOLO V5 model, trained for facial eye region detection, proved to be an effective tool and served as a critical step in the proposed AI diagnostic system.

### 2.3 Model setting for TAO activity sign prediction

In our EnsembleDL system, we partitioned all images into training and testing sets in an 8:2 ratio. Prior to being input into the model, each image was resized to 224 × 224 pixels with random data augmentation of the crop and horizontal flip. Model development was implemented using the PyTorch framework with a learning rate of 2 × 10^−4^. We employed Adam optimizer Kingma and Ba (24) with a weight_decay of 5 × 10^−4^ for the model optimization. Training was performed on an NVIDIA Geforce 2080TI GPU with a batch size of 8 and maximum epoch number of 100. This setup ensured optimal learning and accuracy in our predictive tasks by leveraging the power of ensemble learning to handle multi-view and multimodal data.

### 2.4 Ensemble deep learning system for CAS determination

#### 2.4.1 Model architecture

The EnsembleDL system developed in this study innovatively integrates multiple views and modalities that are specifically designed to address the multifaceted nature of TAO activity. These modalities encompass two distinct types of images: anterior segment slit-lamp photographs and facial images. The anterior segment slit-lamp photographs captured various perspectives of each eye, including frontal and lateral gaze (left and right) views. For the patients’ facial images, we incorporated views from five different directions: up, down, left, right, and primary positions.

#### 2.4.2 Feature extraction

In our EnsembleDL system, each image *X_v,m_*—where *m* denotes the modality (anterior segment slit-lamp photograph or facial image) and *v* represents the specific view (e.g., left eye frontal or right eye right gaze)—is processed through a shared ResNet50 network He et al. (25). This network acts as a feature extractor and is known for its robust performance in image classification tasks. The features extracted from each view of each modality are represented by *F_v,m_*, as shown in Equation 1.


(1) $$F_{v,m}=ResNet50\left(X_{v,m}\right)$$

#### 2.4.3 Feature concatenation

After extracting features from each view and modality, we concatenated them to form a comprehensive feature set *F*, as shown in Equation 2. This concatenation integrated the diverse characteristics captured from each perspective, forming a rich multidimensional representation of TAO activity.


(2) $$F=Concat\left(F_{1,1},F_{2,1},\mathrm{...},F_{V,\;M}\right)$$

In this formulation, *V* and *M* denote the total numbers of views and modalities, respectively.

#### 2.4.4 Sign prediction

The final step of our model is the classification of key signs of TAO activity. For learned feature *F_i_* from the *i*th patient, we utilized a cross-entropy loss function Shannon (26) to effectively train the EnsembleDL model to distinguish the various signs of TAO activity, as shown in Equation 3:


(3) $$L\;=\;-\sum_{i=1}^{N}\;y_{i}\;log\left(P\left(F_{i}\right)\right)$$

where *L* is the loss function, *N* represents the number of patients, *y_i_* is the indicator variable for the true labels in each sign, and *P* is the prediction layer for the learned multi-view multimodal features.

Through the above framework, this EnsembleDL approach not only facilitates nuanced analysis of TAO activity but also enhances the prediction accuracy of CAS.

### 2.5 CAS calculation and grading model

#### 2.5.1 Predictive modeling for TAO signs

Utilizing our multi-view, multimodal deep learning model, we first predicted key signs of TAO activity for each patient. These signs include eyelid redness and swelling of the eyelids, conjunctiva, and caruncle or plica Goldstein et al. (27), Dickinson and Perros (28). The prediction was based on the analysis of multiple anterior segment slit-lamp photographs and facial images of each patient. We accurately identified the presence of these signs using the previously described ResNet-based prediction model.

#### 2.5.2 Incorporating subjective symptoms

Alongside the objective signs predicted by the model, we also considered patient-reported subjective symptoms, specifically spontaneous retrobulbar pain and pain on attempted upward or downward gaze. These subjective symptoms play a crucial role in determining the overall CAS.

#### 2.5.3 CAS formulation

The CAS for each patient was calculated by summing 1 score each for the presence of the five predicted TAO activity signs and two subjective symptoms, resulting in a total score that ranges from 0 to 7. Mathematically, the CAS *S* can be formulated by Equation 4:


(4) $$S=\sum_{i=1}^{5}\;P_{i}+SRP+AGP$$

where *P_i_* represents the presence (1) or absence (0) of each of the five TAO signs predicted by the model, *SRP* denotes spontaneous retrobulbar pain, and *AGP* represents pain on attempted upward or downward gaze.

#### 2.5.4 CAS grading

Based on the calculated CAS, we classified patients into two categories: active and inactive TAO Kampmann et al. (29), Karhanová et al. (30). Patients with CAS of 3 or higher (*S* ≥ 3) were categorized as having active TAO, whereas those with a score smaller than 3 (*S<* 3) were classified as having inactive TAO. This categorization is crucial for guiding the clinical management and treatment of patients with TAO.

## 3 Results

### 3.1 Data characteristics

#### 3.1.1 Patient cohort and demographics

We included 156 patients from Shanxi Eye Hospital Affiliated to Shanxi Medical University, and reviewed their records collected from May 2020 to September 2023. The average age of the patients was 49.2 years, with female representation of 47.4% (74/156 patients). All patients underwent comprehensive TAO examinations, including anterior segment slit-lamp photographs and facial images, and data on medical history, systemic diseases, vision, intraocular pressure, color vision, eye protrusion, eye movement, and degree of eyelid retraction were also obtained.

#### 3.1.2 Clinical characteristics

The average CAS among the patients was 2.2. The most common symptoms were eyelid swelling and redness of the conjunctiva. Patients who presented with active TAO (CAS≥3) accounted for 46.2% (72/156) of the cohort, whereas 13.5% (21/156) had highly active TAO (CAS≥5). The patient distributions for each sign are presented in **Table 1**, and the distribution of CAS scores are summarized in **Table 2**.

**Table 1:** The patient distributions for each sign of TAO activity sign.

Signs	Positive	Negative
RE	39	117
SE	98	58
RC	94	62
SC	27	129
SCP	53	103

TAO, thyroid-associated ophthalmopathy; RE, Redness of Eyelids; SE, Swelling of Eyelids; RC, Redness of Conjunctiva; SC, swelling of conjunctiva; SCP, Swelling of Caruncle or Plica.

**Table 2:** The distribution of CAS scores.

CAS Score	0	1	2	3	4	5	6	7
Number of Patients	54	7	23	32	19	11	6	4

### 3.2 The diagnostic performance of signs of TAO activity

#### 3.2.1 Overall performance

Extensive experiments were conducted to validate the predictive capabilities of the proposed multi-view multimodal EnsembleDL model for TAO activity signs and its effectiveness in calculating CAS. To comprehensively assess the performance of the model, we calculated a range of metrics, including accuracy, specificity, precision, recall, and F1-score Chetoui and Akhloufi (31), Yang et al. (32). These metrics provide detailed insights into the model’s ability to accurately identify TAO symptoms and categorize active cases.

The experimental results summarized in **Table 3** reveal the efficacy of our multi-view multimodal EnsembleDL in diagnosing the key signs of TAO activity. For Redness of Eyelids (RE), the model attained a respectable accuracy of 75.0% and high specificity of 95.0%; however, the low recall of 41.7% suggests room for improvement in identifying all positive cases, as reflected by the F1-score of 56.0%. In contrast, Swelling of Eyelids (SE) and Redness of Conjunctiva (RC) exhibited remarkable predictive accuracy, with each achieving an accuracy of 93.8% and similarly high precision, recall, and F1-scores, indicating a balanced and effective performance. Swelling of Conjunctiva (SC) achieved a moderate accuracy of 84.4% with slightly lower precision and recall, suggesting areas for refinement in future model iterations. Exceptionally, Swelling of Caruncle or Plica (SCP) demonstrated outstanding accuracy and precision, both at 93.8%, with an impressive area under the curve (AUC) of 99.6%, highlighting the robust diagnostic capability of the model for this sign.

**Table 3:** The prediction performance of the key signs of TAO activity.

Signs	Accuracy	Specificity	Precision	Recall	F1-score	AUC
RE	0.750	0.950	0.833	0.417	0.560	0.867
SE	0.938	0.833	0.962	0.962	0.962	0.974
RC	0.938	0.889	0.956	0.956	0.956	0.981
SC	0.844	0.905	0.800	0.727	0.762	0.745
SCP	0.938	0.929	0.945	0.945	0.945	0.996

TAO, thyroid-associated ophthalmopathy; RE, Redness of Eyelids; SE, Swelling of Eyelids; RC, Redness of Conjunctiva; SC, Swelling of Conjunctiva; SCP, Swelling of Caruncle or Plica.

The model exhibited a high level of precision and accuracy in identifying various signs of TAO activity, particularly SE, RC, and SCP, while indicating potential areas for enhancement of RE and SC predictions.

#### 3.2.2 Visualization

**Figure 3:**
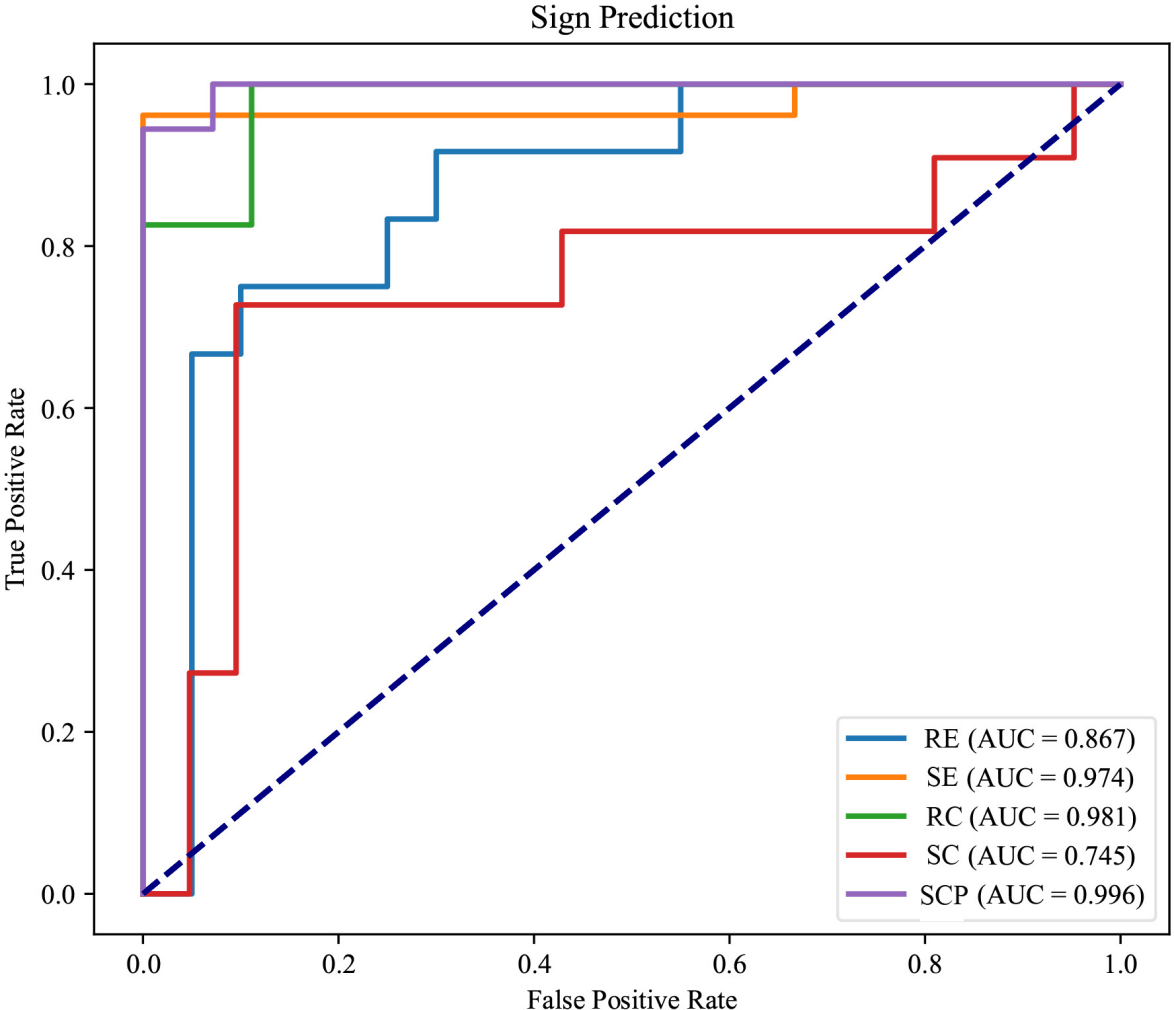
The receiver operating characteristic curves of each TAO sign prediction task of the model.

The receiver operating characteristic (ROC) curves Zweig and Campbell (33) presented in **Figure 3** offer a comprehensive assessment of the diagnostic process of our multi-view multimodal EnsembleDL model across a spectrum of TAO activity signs. For SE and RC, the AUCs were impressively high (0.974 and 0.981, respectively), reflecting a level of predictive accuracy that speaks to the model’s robust capabilities. The high precision and recall of these signs demonstrate the model’s ability to accurately discern the nuanced manifestations of TAO activity with remarkable reliability.

In the analysis of RE, an AUC of 0.867, although respectable, indicated room for growth. The curve does not ascend steeply toward the upper left corner as its counterparts do, underscoring the disparity in the model’s sensitivity to this particular sign. This observation is echoed by a recall that lags behind, suggesting the necessity of further refining the model to enhance its sensitivity for RE.

Regarding SC, the modest AUC of 0.745 indicates a more pronounced need for optimization. The main reason for this low AUC is that the positive samples are significantly fewer than the negative cases (an imbalanced distribution). Here, the model’s performance highlights a dichotomy; although capable, it does not reach the high standard set by the predictions for SE and RC. The middling accuracy and precision of SC prediction imply an opportunity to improve the model’s discernment abilities, potentially by refining the algorithms or enriching the training data to capture a broader representation of SC cases.

Conversely, the ROC curve for SCP was characterized by an exceptional AUC of 0.996. This denotes not just a return to form but also a transcendent performance that sets a benchmark for the model’s diagnostic accuracy. The substantial AUC for SCP signifies a near-perfect ability to differentiate between positive and negative instances of this sign, re-affirming the model’s ability to recognize more overt TAO symptoms.

**Figure 4:**
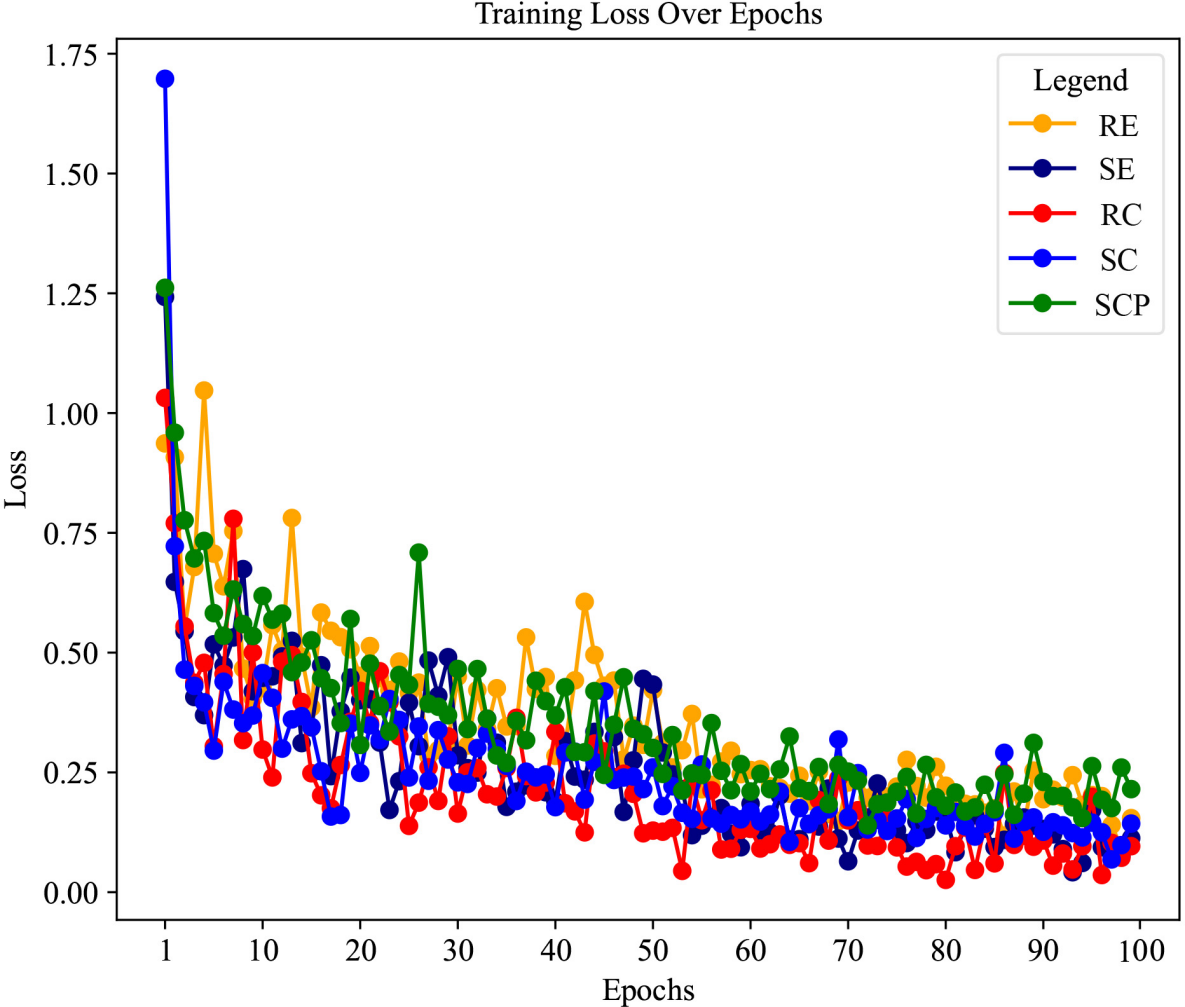
The loss curves of each thyroid-associated ophthalmopathy sign prediction task of the model in the training stage.

In addition, the loss curves depicted in **Figure 4** show the collective training progression of our multi-view multimodal EnsembleDL model for TAO sign prediction tasks. Each curve, corresponding to a sign, converged to a lower loss, demonstrating the capacity of the model to adapt and improve its prediction accuracy over epochs. A sharp initial decline across board signifies a robust learning rate, particularly for signs such as SE and RC, which correlates with high AUC values and reflects swift learning in the early stages. As the epochs advanced, the curves tended to plateau, indicating that the model achieved a stable level of loss minimization—a testament to its ability to efficiently capture diagnostic patterns of TAO activity.

As revealed by the loss curves, the overall performance of the model illustrates a successful learning process, and the subtle differences between the various signs highlight the complexity of the task. The varying rates of convergence across the curves suggest that certain signs of TAO activity, such as SCP, are modeled with higher accuracy and confidence, as evidenced by their steep and steady loss decline. However, the more gradual decrease in the loss of RE and variability seen in SC point to areas where the model may benefit from further tuning. These insights provide a roadmap for future refinement of the model, ensuring ongoing improvements and steering the development toward a robust, comprehensive diagnostic tool for TAO activity.

While the model demonstrated a commendable capacity for predicting signs of TAO activity, with SE, RC, and SCP detection being particularly distinguished, the subtle disparities in RE and SC highlight the ongoing challenge of developing a universally robust AI system. These insights direct us toward targeted enhancements in future iterations, ensuring continuous evolution of the model’s sophistication and a steadfast commitment to clinical excellence.

### 3.3 CAS scoring and grading performance

The predictive performance of our deep learning system for classifying TAO into active and inactive cases based on the CAS is presented in **Table 4**. Our model achieved high accuracy (0.906) across all three evaluation metrics: the weighted, macro-, and micro-averaging methods. This demonstrates the consistent performance of the model in recognizing active TAO (CAS≥ 3) versus inactive cases (CAS< 3). Specifically, the model’s precision and recall were both high for the weighted and micro measures, indicating reliable predictability across the spectrum of CAS assessment.

**Table 4:** The prediction performance for active TAO based on CAS, including active(CAS≥3) and inactive (CAS*<*3) cases.

Types	Accuracy	Specificity	Precision	Recall	F1-score
Weighted	0.906	0.833	0.906	0.906	0.906
Macro	0.906	0.833	0.907	0.892	0.898
Micro	0.906	0.833	0.906	0.906	0.906

**Figure 5:**
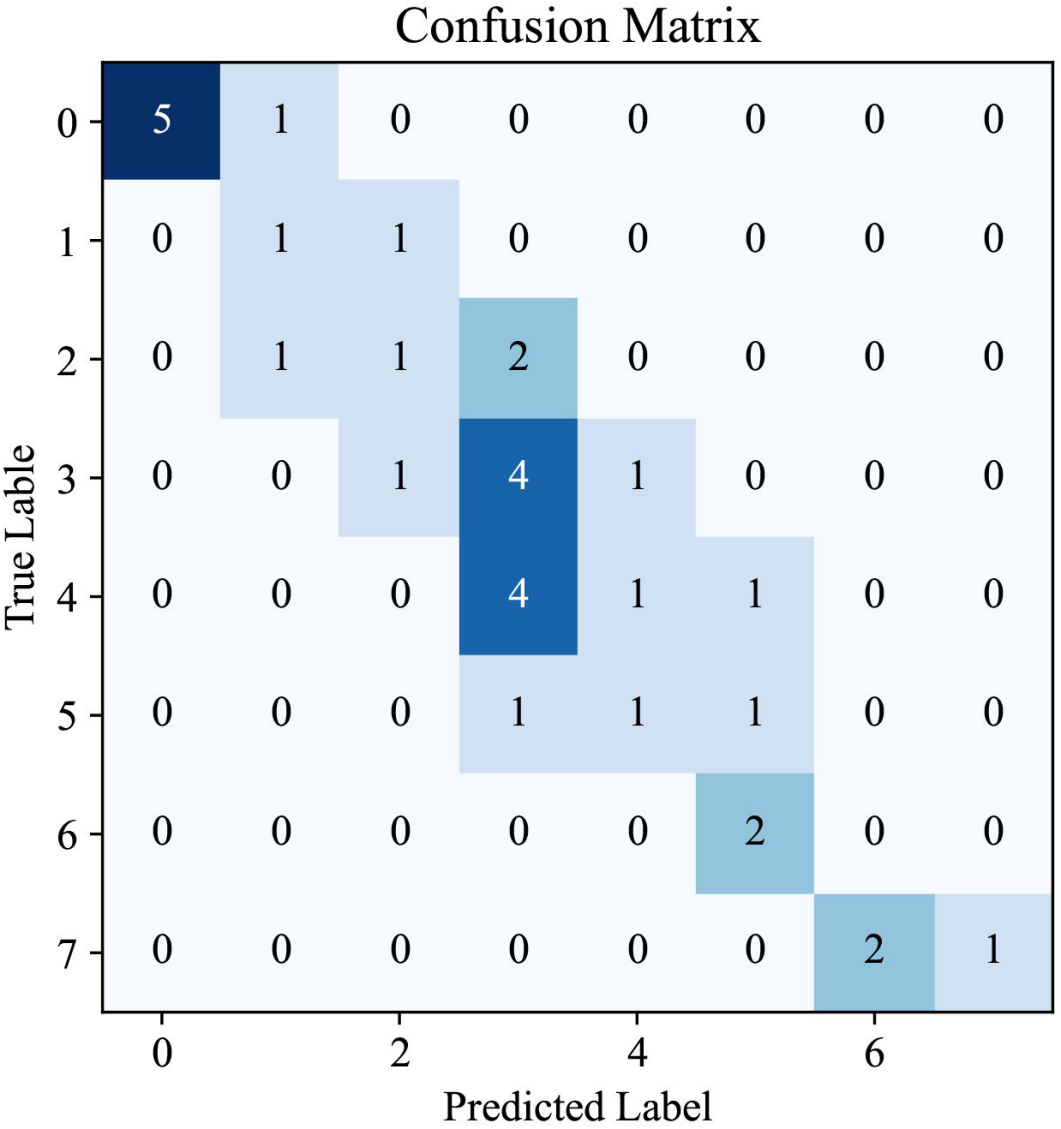
The confusion matrix of Clinical Activity Score prediction from our AI system.

The confusion matrix provided in **Figure 5** offers a visual representation of the performance of our AI system in predicting CAS for TAO. This matrix is an essential tool for understanding the distribution of the model predictions in relation to true labels, offering an in-depth perspective on the accuracy of CAS determination.

Analysis of the matrix revealed a high degree of accuracy for certain scores, as indicated by the concentration of higher values along the diagonal, which represent correct predictions. Notably, the model showed a strong ability to accurately identify cases with CAS of 0, suggesting a robust capability for recognizing patients with inactive TAO. Similarly, the model performed well in correctly predicting higher CAS values, which are critical for identifying patients with more severe TAO manifestations. However, the presence of non-zero values off the diagonal indicates instances in which the model predictions deviated from the true labels. These discrepancies are most apparent in the middle range of the CAS spectrum, highlighting a potential area for improvement in the ability of the model to differentiate between adjacent CAS categories.

The pattern observed in the confusion matrix underscores the effectiveness of our AI system in classifying TAO activity into appropriate CAS categories. It also indicates opportunities for further refinement, particularly in improving the precision of the model for mid-range values of the CAS. The insights gained from this confusion matrix are invaluable for directing future enhancements to the model, with the ultimate goal of achieving a nuanced and precise tool for TAO diagnosis and management.

### 3.4 Comparison with single-view unimodal setting

**Figure 6:**
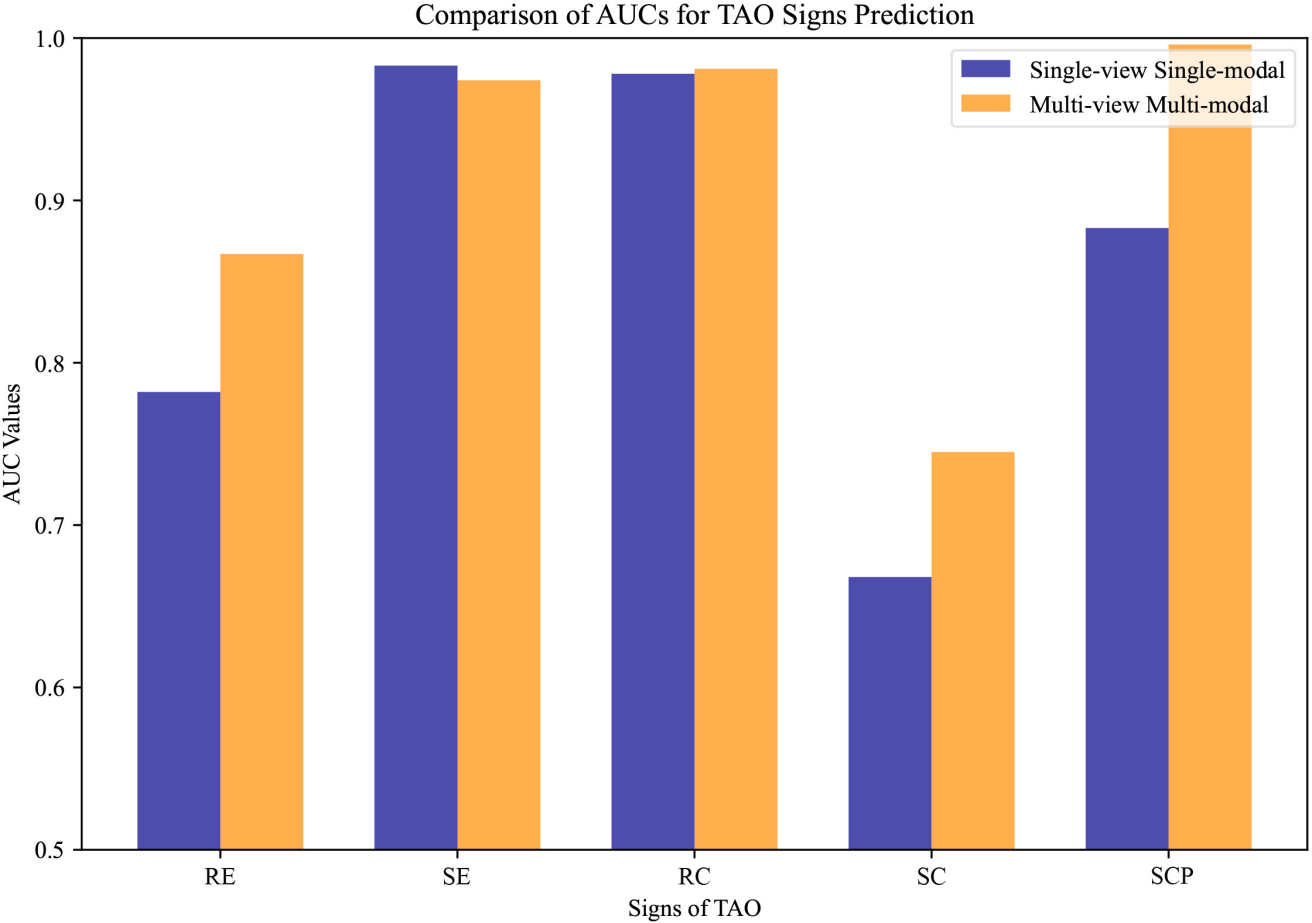
The compared area under the curve values of thyroid-associated ophthalmopathy activity sign prediction under different settings. Single-view unimodal setting denotes that the sign was predicted from only a primary-positioned facial image, while the multi-view multimodal one represents our proposed sign prediction model based on multiple angles from both anterior segment slit-lamp photographs and facial images.

To validate the superiority of our multi-view multimodal EnsembleDL system over traditional single-view analyses, a comparative experiment was conducted. The experiment involved training our sign prediction model solely on primary-positioned facial images. The results are presented in **Table 5**, and **Figure 6** shows the advancements achieved by our multi-view multimodal approach.

**Table 5:** The prediction performance for the key signs of TAO under single-view unimodal setting.

Signs	Accuracy	Specificity	Precision	Recall	F1-score	AUC
RE	0.688	0.818	0.5	0.4	0.444	0.782
SE	0.875	0.917	0.944	0.85	0.895	0.983
RC	0.906	1	1	0.857	0.923	0.978
SC	0.625	0.737	0.545	0.462	0.5	0.668
SCP	0.813	0.917	0.938	0.75	0.833	0.883

TAO, thyroid-associated ophthalmopathy; AUC, area under the curve; RE, Redness of Eyelids; SE, Swelling of Eyelids; RC, Redness of Conjunctiva; SC, Swelling of Conjunctiva; SCP, Swelling of Caruncle or Plica.

The results demonstrate that the innovative multi-view multimodal framework of our AI system has significant improvements in the diagnostic accuracy for TAO activity signs compared with the single-view unimodal setting that relies solely on primary-positioned facial images. As illustrated in **Figure 6**, our model consistently achieved higher AUC values across most signs, indicating a more comprehensive analysis capability. However, RC showed a subtle gain, while SE expressed little weakness.

The contrast is particularly evident in the cases of RE, SC, and SCP, where the multi-view multimodal system outperforms the single-view unimodal setting by a significant margin. This indicates the added value of incorporating multiple photographic angles and anterior segment slit-lamp photographs, which enhanced the capability of the model to discern the complex interplay of signs present in TAO.

**Figure 7:**
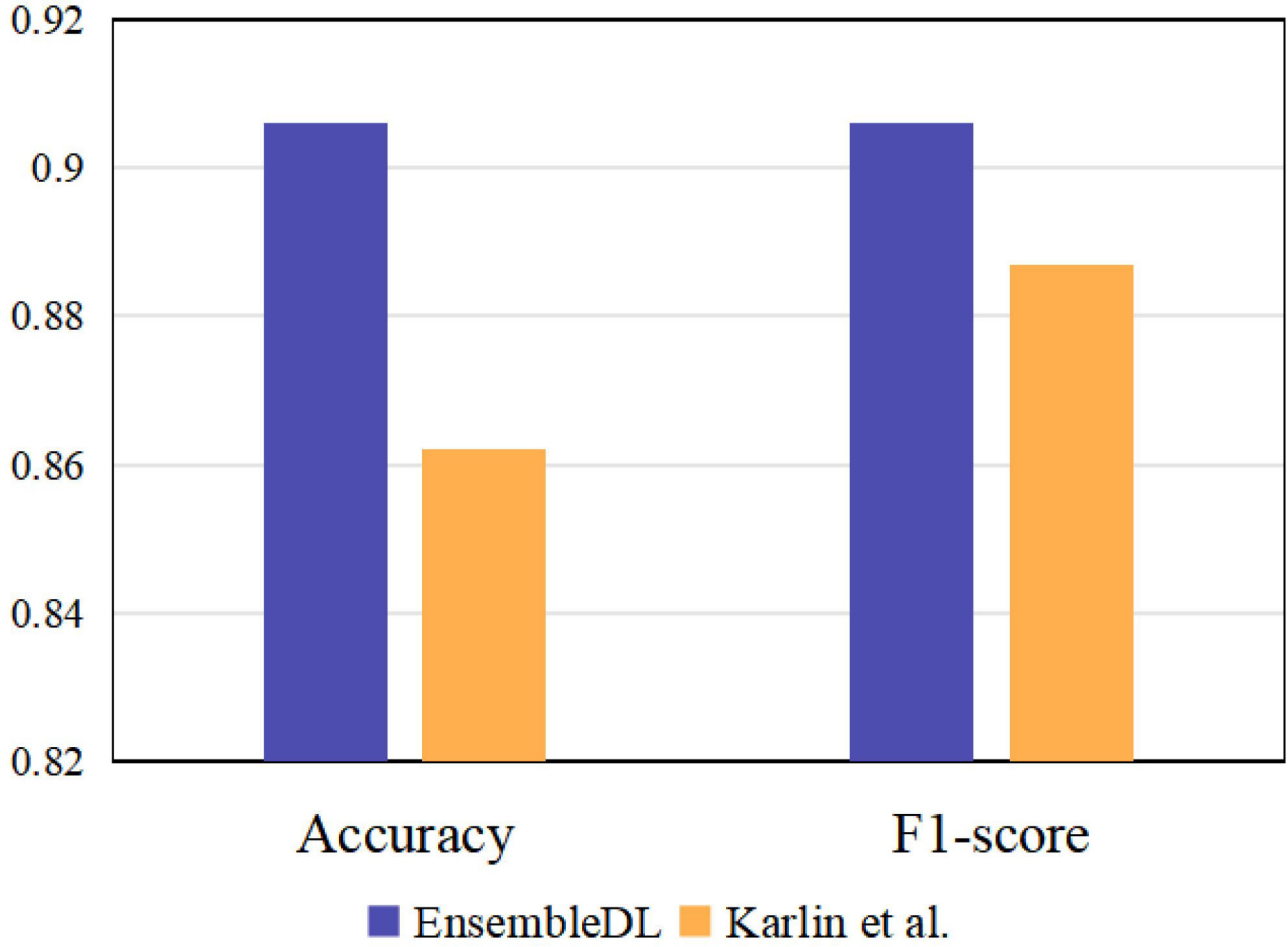
The comparison between our EnsembleDL and existing single-view unimodal setting work Karlin et al. (14).

To further reveal the superiority of our EnsembleDL, we introduce an existing single-view unimodal TAO diagnosis model to compare, e.g. Karlin et al. (14), which utilized ResNet18 to predict the TAO signs. We implement ResNet18 network under single-view unimodal setting on our dataset by predicting active TAO, and the comparison are shown in **Figure 7**. The results report our multi-view multimodal EnsembleDL system surpass the Karlin et al. (14) with a large margin in accuracy and F1-score, demonstrating the effectiveness of our proposed EnsembleDL system.

Furthermore, the consistency of the multi-view multimodal approach with clinical diagnostics is highlighted by its ability to closely mimic the comprehensive decision-making processes of clinicians. This alignment with clinical practice is paramount, as it ensures that the model’s predictions are not only statistically robust, but also practically relevant and clinically interpretable.

## 4 Discussion and conclusion

### 4.1 Discussion

TAO is the most common extrathyroidal manifestation of GD, with an occurrence rate of 25–40% in patients with GD Bartalena et al. (1). It is also observed in 2% of patients with chronic lymphocytic thyroiditis, a minority of patients with hypothyroidism, and some individuals with normal thyroid function. GD is a common disease in endocrinology. The relationship between the onset of GD and TAO is such that 43% of patients present concurrently, whereas GD precedes TAO in 44% of cases. Most patients with TAO first present to the endocrinology department, where many physicians lack experience in diagnosing early TAO. An experienced ophthalmologist is required for diagnosis; however, the current number of ophthalmologists specializing in orbital diseases in China is insufficient and does not meet this demand.

In this study, we developed an EnsembleDL system that combines anterior segment slit-lamp photographs and facial images to assess and detect the activity of TAO, and the system demonstrated robust performance. With the integration of anterior segment slit-lamp photographs, subtle inflammatory changes were captured well, enabling the system to identify inflammatory changes in patients with early-stage TAO. The treatment plan for TAO is contingent on the evaluation of its activity and severity, and early assessment of activity is particularly crucial. Proactive anti-inflammatory or immunosuppressive treatments, if initiated in the early stage, can effectively control the disease and prevent its progression to severe stages Bartalena et al. (3).

The innovative methodology proposed in this study integrates a multi-view multimodal deep learning model with residual networks, providing significant advancement in the predictive modeling of TAO activity signs. In contrast to conventional approaches, our study pioneers the use of an eye object detection network achieved through YOLO v5, which incorporates feature fusion across various perspectives of anterior segment slit-lamp photographs and facial eye images to extract overall multi-view multimodal representations. Subsequently, our method generated final predictions for the five key signs of TAO and computed the CAS in conjunction with patient-reported spontaneous retrobulbar pain and pain experienced during attempted upward or downward gaze. The superior performance of our model over traditional single-view unimodal methods in predicting individual TAO symptoms and calculating CAS underscores its efficacy. Our experimental results confirm the exceptional precision and accuracy of our approach, highlighting its potential for enhancing clinical treatment decision-making.

To demonstrate its superiority, we compared the performance of our EnsembleDL model, which utilizes multi-view multimodal data, to the results of single-view unimodal systems, as in previous studies. Our model demonstrated significant superiority for the diagnosis of TOA. Specifically, for RE, the accuracy of the ensemble model reached 75.0% compared to 68.8% of the single-view unimodal model. More impressively, the recall increased from 40.0% to 41.7%, and the F1-score improved from 44.4% to 56.0%, with an AUC of 0.867 versus 0.782 in the single-view unimodal system.

Meanwhile, the model performance in diagnosing RC and SCP was significantly improved, with the accuracy increasing from 90.6% and 81.3% respectively in the single-view unimodal settings to 93.8% for both signs in the multi-view multimodal system of EnsembleDL. The AUC also improved from 0.978 and 0.883 to 0.981 and 0.996 for RC and SCP respectively. These results suggest that integrating different views and modalities can more accurately capture subtle changes in patients’ conditions, which are crucial for diagnosing TAO activity. These findings substantiate the advantage of our EnsembleDL model: integrating multi-view and multimodal data for predicting TAO activity and calculating the CAS provides more comprehensive and detailed data for the model to learn from and make predictions. The performance of our model validates the importance of multi-view and multimodal data in deep learning models for medical diagnosis.

In the evaluation of Redness of Eyelids (RE), our ensemble model demonstrated a moderate decrease in performance metrics, with an accuracy of 0.750, specificity of 0.950, and a notably lower recall of 0.417. Several factors may account for this diminished accuracy. Variegated clinical presentations of eyelid redness can lead to substantial interpretive variability, even among expert clinicians. The model’s ability to discern subtle gradations of redness is also likely impeded by inconsistent lighting, variability in skin tones among patients, and the presence of makeup. The challenge is further compounded by the model’s need to differentiate between mild inflammatory signs and normal physiological variations. Although high specificity suggests effective discrimination of the absence of RE, the lower sensitivity indicates the necessity to robustly identify true RE manifestations. Future model refinements will aim to better capture these nuances by expanding image data diversity, improving preprocessing routines, and deploying more sophisticated neural architectures to enhance sensitivity without compromising specificity.

Our approach not only refines the prediction of TAO activity signs with high fidelity, but also innovates the computation of CAS by incorporating a fusion of objective signs and subjective symptoms, culminating in a comprehensive system that promises to advance clinical assessment and management of TAO based on disease activity. Moreover, this deep learning system proved to be effective in the critical task of stratifying TAO into active and inactive categories. The comprehensive evaluation metrics of the model underscore its effectiveness in identifying TAO symptoms, with certain areas identified as potential areas for improvement in future iterations. The convergence of high accuracy, precision, and recall across the different averaging methods highlights the potential of the system to serve as a valuable adjunct in clinical settings. From the results obtained in this study, our AI system not only aligns with the clinical diagnostic process, but also extends its capabilities by providing multidimensional analysis of TAO activity signs. This approach indicates the potential of the model in clinical settings, where accurate diagnosis is critical for effective patient management and treatment outcomes. The integration of multiple views and modalities, as substantiated by our findings, is imperative for advancing the field of AI-assisted diagnostics and re-affirms our commitment to enhancing patient care through innovation.

The popularity of smartphones and portable slit lamps can help endocrinologists and doctors in primary hospitals to obtain patients’ facial images and anterior segment slit lamp photos. The EnsembleDL system can provide feedback on TAO activity in a timely manner so that patients can be promptly referred to ophthalmologists for more effective diagnosis and treatment. Moreover, the proposed EnsembleDL can be built into a WeChat mini program, and patients can log in through their smartphones and take photos according to the requirements. The system allows preliminary detection of activity, and it is recommended that patients with active TAO visit an ophthalmology clinic for active treatment.

The multi-view multimodal diagnostic model presented in this paper has significant implications for future research. Its capacity to provide a comprehensive analysis of TAO could lead to the development of more personalized treatment plans, improve patient management, and potentially contribute to better understanding of the pathophysiology of TAO. The framework established herein paves the way for further innovations in AI-assisted diagnostics, potentially expanding to other complex diseases that require a similar multidimensional approach for accurate diagnosis.

Recognizing the constraints in clinical practice, where endocrinologists may not routinely utilize slit lamps, we envisage the development of a bespoke imaging apparatus. This device, harmonizing the functionalities of a slit lamp with an optimized facial image capture system, will be specifically tailored to complement our EnsembleDL model. By designing a standardized protocol for image acquisition across the different modalities, we aim to bolster the system’s robustness and extend its applicability. Future research endeavors will prioritize the refinement of this integrated imaging solution, aligning closely with the EnsembleDL’s capabilities to ensure seamless and efficient diagnostic workflows.

### 4.2 Conclusion

In conclusion, our study proposes a novel and effective multi-view multimodal deep learning system for assessing TAO activity. The EnsembleDL system integrates anterior-segment slit-lamp photos and facial images for improved performance. The diagnostic accuracy of our AI diagnostic model for TAO activity was significantly better than that of the AI diagnostic model that used only facial images, and our model could intelligently and objectively evaluate the activity of TAO. The performance of the system is likely to improve further as data continue to be supplemented. This system can help endocrinologists and primary care physicians to identify the need for referral and is expected to enable patients to monitor their disease activity and consult a doctor in a timely manner. Using this system to screen the activity of TAO and promptly refer patients for treatment will help clinicians to make diagnostic and treatment decisions, and improve the quality of life and prognosis of patients with TAO.

### 4.3 Limitations

Notwithstanding the robust performance of the model, we acknowledge the limitations of this study. The model’s performance, although superior, is contingent on the quality and variety of the data on which it has been trained. Future research could benefit from a larger and more diverse dataset, encompassing a broader spectrum of CAS for TAO and patients’ demographics. Additionally, longitudinal studies assessing the predictive power of the model over time are invaluable for confirming its clinical utility and robustness. Despite these limitations, our study represents a substantial leap in the application of AI in ophthalmology, and sets a new benchmark for future endeavors in this rapidly evolving field.

## Data availability statement

The raw data supporting the conclusions of this article will be made available by the authors, without undue reservation.

## Ethics statement

Owing to the retrospective design of the study and the use of past medical records and facial images, the ethics review committee waived the requirement for informed consent.

## Author contributions

CY: Conceptualization, Data curation, Formal analysis, Investigation, Methodology, Writing – original draft, Writing – review & editing. ZZ: Conceptualization, Data curation, Investigation, Methodology, Writing – original draft. GZ: Data curation, Formal analysis, Methodology, Visualization, Writing – original draft. HL: Investigation, Visualization, Writing – original draft. RZ: Data curation, Formal analysis, Writing – original draft. GL: Investigation, Methodology, Writing – original draft. JR: Formal analysis, Methodology, Writing – original draft. WY: Conceptualization, Methodology, Writing – review & editing. BS: Conceptualization, Funding acquisition, Investigation, Supervision, Writing – review & editing.
